# Electricity consumption data of a middle-income household in Gauteng, South Africa: Pre and Post COVID-19 lockdown (2019-2021)

**DOI:** 10.1016/j.dib.2022.108341

**Published:** 2022-06-01

**Authors:** R.O. Masebinu, N. Kambule

**Affiliations:** Department of Geography, Environmental Management, and Energy Studies, Faculty of Science, University of Johannesburg, South Africa

**Keywords:** Hourly electricity consumption, Real-time data, Residential building, Energy efficiency, Data analysis, COVID-19

## Abstract

The real-time hourly electricity consumption data of a middle-income household in the Gauteng Province of South Africa was tracked for 30 months (i.e. 2019 to 2021) over three different residential properties. Layout diagram and physical characteristics of each of the residential properties are provided. An energy audit of all appliances at the residence was conducted at the beginning of the study and acquisition of new appliances was also captured. The aggregated electricity consumption throughout the study of all appliances at the family residence was captured from a single-phase electricity distribution sub-panel. The granularity of the captured data was at the hourly resolution level and presented as kilowatt-hour. A total of 20,852 hours of data points were captured. The data has not been processed further. In addition to the energy consumption data, 16 months of hourly data for wind speed, temperature, and humidity of the closest weather station to two of the residential properties has been provided. The energy consumption data will be useful for teaching and research in energy consumption prediction studies, and energy management strategy development. Considering the timing of the study that encompasses pre-COVID-19 and three peaks of COVID-19 in South Africa, the data can be useful in analysing the impact of COVID-19 on household electricity consumption.

## Specifications Table

**Table:** 

Subject	Energy
Specific subject area	Residential electricity consumption
Type of data	Table
How data were acquired	The data were acquired using an Efergy Engage Hub Kit. The instrument has a 98% measurement accuracy, according to the manufacturer specification sheet.
Data format	Raw
Description of data collection	A current transformer was attached to a single-phase conductor wire supplying the family residence with electricity. The current transformers were connected to a single data transmitting hub. A data receiver collects the data automatically from the transmitting hub and uploads it to the cloud. An Efergy Engage App was used to visualise the data and download the raw data directly from the cloud server. An additional tabletop datalogger that does not require internet connectivity was also used to collect the electricity consumption data.
Data source location	Institution: University of Johannesburg Region: Gauteng Country: South Africa Latitude and longitude for collected data: 26.1836° S, 27.9977° E
Data accessibility	Repository name: Mendeley DataData identification number: 10.17632/39fdmmv28j.1Direct URL to data: https://data.mendeley.com/datasets/39fdmmv28j/draft?a=42b9ba69-db6a-480b-bda8-ad8cd68e7c04Instructions for accessing these data: The data does not have any access restriction.

## Value of the Data

• In sub-Saharan Africa, the lack of real-time electricity consumption data during the design and sizing of renewable energy technology systems make variability between simulated and real system performance differ significantly [1]. The data presented is useful in residential apartments electrification planning where real-world variability is required.
• COVID-19 has changed the dynamics of household load profile [2]. During COVID-19 residential properties served as business spaces and other productive energy use alongside their use as homes. The data presented will be useful in assessing changes in household energy consumption before, during and after the three major waves of COVID-19 as experienced in South Africa.
• The data would be of benefit to data scientists, energy building researchers and researchers studying the impact of COVID-19 induced restrictions on residential sector electricity consumption. The hourly time series data would be of benefit to scientists building electricity demand predictive models with machine learning algorithms.
• The data contains missing data points due to electricity load shedding schedule and other events. When simulating grid-connected energy systems, interruption of the grid is often not accurately modelled in the response strategy. Therefore, the missing data points can be used to predict how an alternative energy system responds at instances when there is an abrupt interruption on grid-supplied electricity.
• The data presented emanates from research work on the role of feedback mechanisms in a middle-income household in South Africa. As such, similar research work can leverage the data presented in this article.
• The data presented contains instances of productive use of energy. Hence, the data presented here can be useful in research that is assessing the changes in electricity consumption profile due to productive use activities at households.

## 1 Data Description

**Fig. 1:**
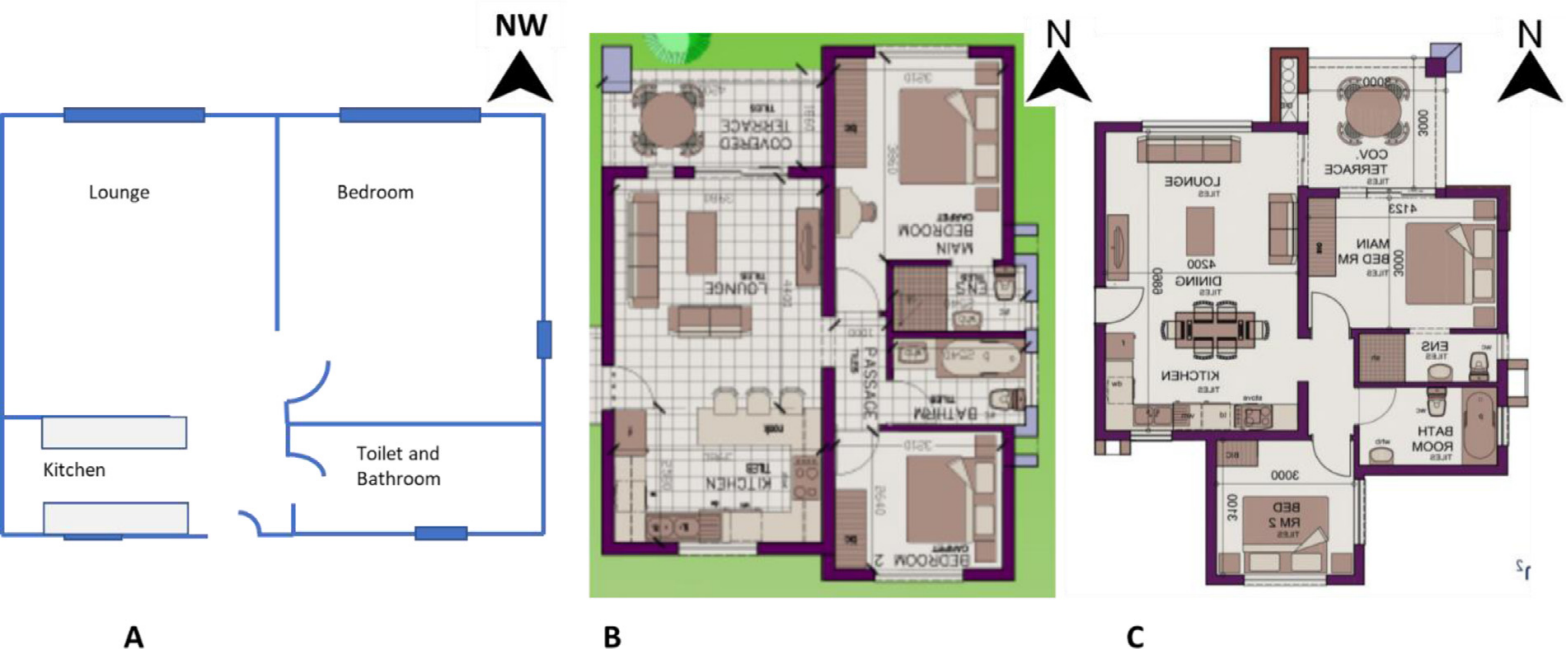
Layout of residential properties.

**Fig. 2:**
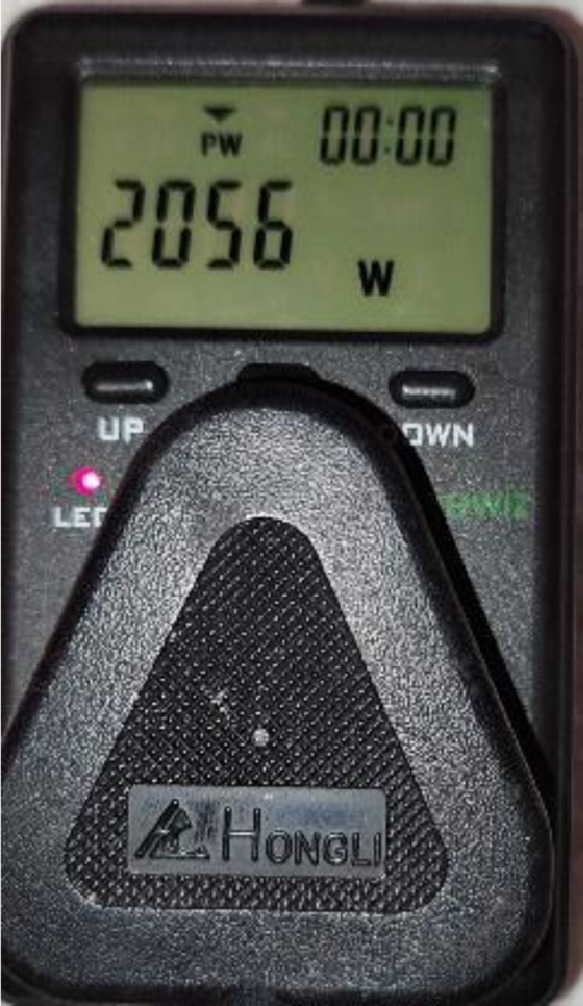
Handheld energy meter.

The electricity consumption data for a middle-income household participating in a research assessing the effect of feedback mechanism on their electricity consumption is presented. The data presented was for the same family at three residential properties spanning over 30 months. The layout of the residential properties is shown in Fig. 1 and their characteristic is presented in Table 1. An energy audit of the household electrical appliances was conducted with a handheld meter as shown in Fig. 2 before the installation of the real-time energy monitor. The summary of the energy audit is presented in Table 2.

**Table 1:** Characteristics of the residential properties.

	Residence A	Residence B	Residence C
**Floor type**	Laminate	Ceramic tiles	Ceramic tiles
**Floor heating**	No	No	No
**Wall type**	Plastered brick wall	Plastered brick wall	Plastered brick wall
**Floor level**	First	Ground	Ground
**Floor size**	40.73 m^2^	74 m^2^	82 m^2^
**Duration of occupation**	01/02/2018-31/01/2020	01/02/2020-31/01/2021	01/02/2021-31/08/2021
**Family size**	Two adults, one baby	Two adults, one toddler	Two adults, one toddler
**Hot water**	Electric kettle	Purchased from an external heat pump.	Purchased from an external heat pump.
**Cooling system**	Natural convection	Natural convection	Natural convection
**Construction date**	Unknown	2017	2019
**Window material**	Single glazed metal frame	Single glazed aluminium frame	Single glazed aluminium frame
**Distance to the weather station**	2 km	11 km	60 km

**Table 2:** Electrical loads of the house residence.

Electrical Appliance	Avg. Daily (kWh/day)	Avg. Weekly (kWh/week)	Comments
Boiling Kettle	0.9180	6.4260	Residence A and used for bathing (Electric geyser was switched off). Residence B and C hot water was purchased from a heat pump.
Tablet	0.0184	0.1290	Residence A, B and C
Reading lamp	0.1413	0.9888	Residence A, B and C
Microwave	0.1177	0.8237	Residence A and B
Router	0.0612	0.4284	Residence A, B and C
Bread Toaster	0.0418	0.2928	Residence A, B and C
Food Blender	0.0011	0.0075	Residence A, B and C
Phone Chargers	0.0251	0.1754	Residence A, B and C
Television	0.1155	0.8083	Residence A, B and C
Refrigerator	1.3006	9.1042	Residence A, B and C
Desktop Monitor	0.0600	0.4200	Residence A, B and C
Laptop 1	0.1673	1.1712	Residence A, B and C
Laptop 2	0.035	0.2449	Residence A, B and C
Lightings	0.0482	0.3373	Residence A, B and C
Flat iron	0.0562	0.3932	Residence A, B and C
Dell Monitor	0.0600	0.42	Residence A, B and C
Washing Machine	0.2790	1.9531	Residence A, B and C
Cooking Stove	2.1880	15.3158	Residence A, B and C, home and productive use
Deep Freezer	0.848	5.90	Residence B and C, home and productive use
Flour Mixer	0.2893	2.0250	Residence B and C, productive use
Deep fryer	0.0286	2.025	Residence B and C, Productive use

**Fig. 3:**
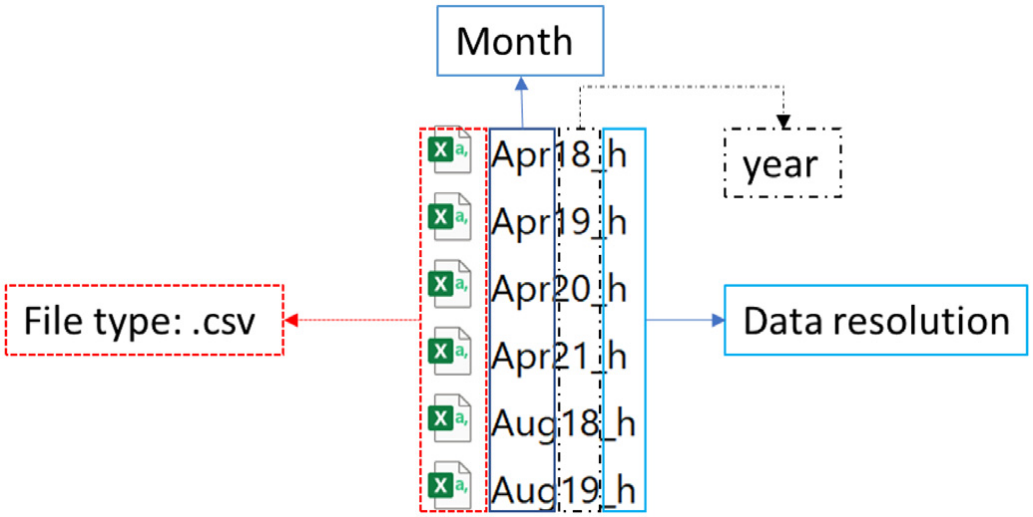
File type and naming convention description.

The electrical energy consumption data presented in this article was gathered using an Efergy real-time energy monitor. Both desktop and cloud-based energy devices were used simultaneously to reduce the tendency of data loss. The power demand is shown in real-time and aggregated hourly to capture the energy consumption in kilowatt-hour. The data was captured from March 2019 to August 2021 consisting of 20,852 hours of data and 1,108-hour points of missing data. A combination of scheduled load-shedding [3,4], interruption in internet connectivity, and relocation of the family during the transition from one property to another contributed to the missing data points. Statistical analysis, data cleaning or segmentation has not been conducted. Hourly electricity consumption for each month is provided as a .csv file and the naming convention is shown in Fig. 3. For example, Mar19_h represents hourly data collected for March 2019 as shown in Table 3. Environmental data for ambient temperature, wind speed and humidity has also been provided. The environmental data covers catchment for two of the residential properties. The third property is approximately 60 km away from the weather station. A sample for ten-day data points over 6 hours is shown in Table 4.

**Table 3:** Sample of energy consumption data and units.

Timestamp	Power (kWh)
01-03-19 0:00	0.05
01-03-19 1:00	0.09
01-03-19 2:00	0.10
01-03-19 3:00	0.06
01-03-19 4:00	0.07
01-03-19 5:00	0.10
01-03-19 6:00	0.26
01-03-19 7:00	0.08
01-03-19 8:00	0.10
01-03-19 9:00	0.27

**Table 4:** Sample of environmental data.

DD	h01	h02	h03	h04	h05	h06
**1**	17.6	17.5	17.5	17.1	16.4	16.0
**2**	18.3	17.8	17.1	17.0	17.2	17.6
**3**	18.3	18.1	17.9	17.4	17.1	17.3
**4**	14.9	14.2	13.9	13.6	13.4	13.4
**5**	18.3	18.7	18.8	17.7	17.2	17.0
**6**	18.5	17.9	17.4	17.3	17.5	17.8
**7**	19.6	18.8	18.2	18.3	17.9	18.0
**8**	17.8	17.3	17.2	17.2	17.1	17.4
**9**	19.7	19.2	19.3	19.3	19.4	19.6
**10**	19.8	18.8	16.9	17.0	17.3	17.7

DD – Day of the Week; h01, h02…h024 is the hour of the day

## 2 Experimental Design, Materials and Methods

**Fig. 4:**
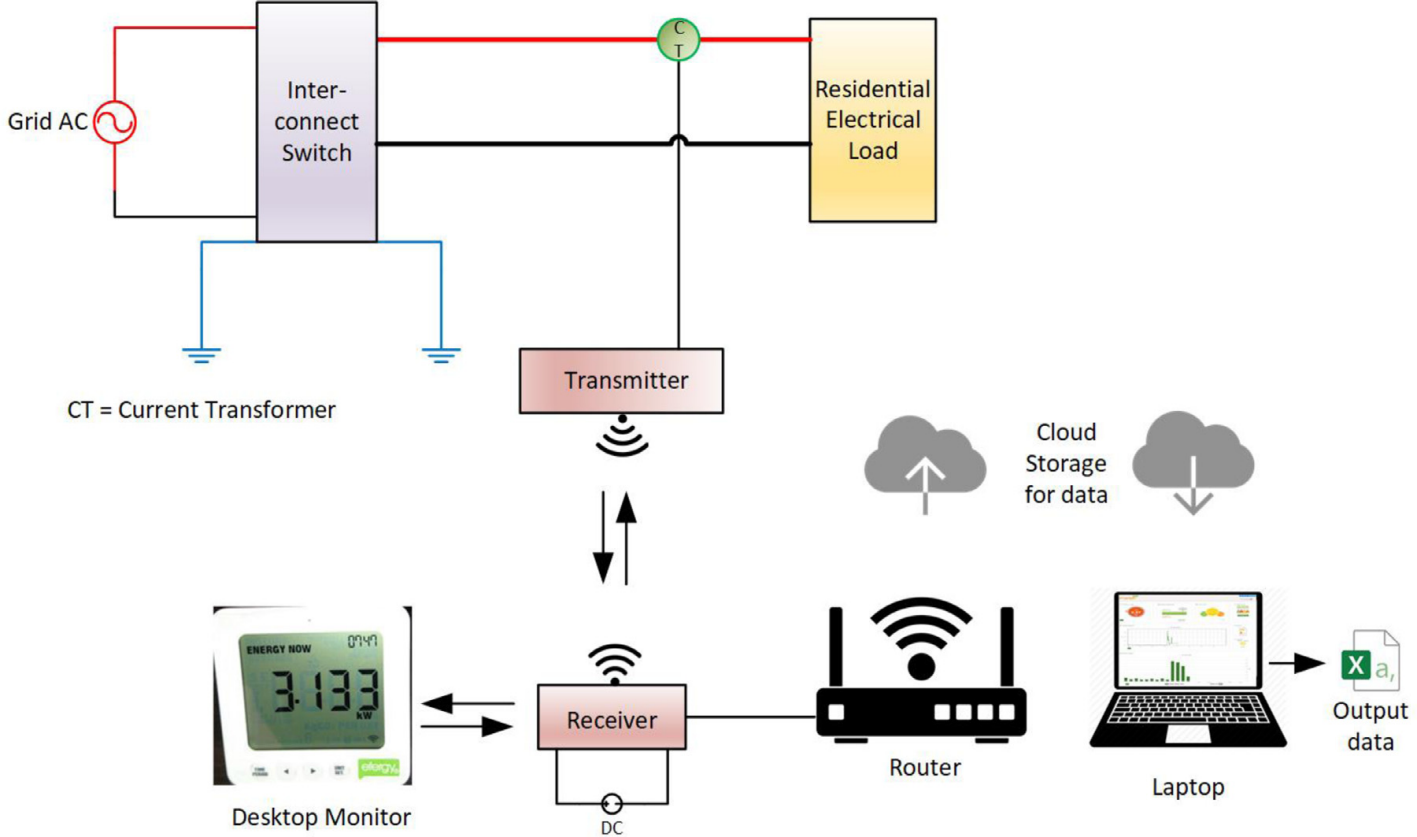
Energy monitoring system layout diagram.

The energy monitoring system consists of a current transformer scanner, a transmitter, an internet-connected receiver and a desktop receiver. The energy monitoring system schematic layout is presented in Fig. 4. The current transformer has a nominal current of 90-120 A, the transmitter has an operating range of 70 m and a receiver (wireless frequency 433.5 MHz, a measurement range of 50 mA to 120 A per phase and a voltage range of 110-300 VAC) [5]. The technical specification of the energy monitoring system hardware is summarised in Table 5.

**Table 5:** Technical specification of the energy monitoring kit hardware.

Components	Current Transformer	Transmitter	Receiver	Desktop Receiver
Conductor diameter	12 mm			
Nominal Current	90-120 A			
Voltage	110-300 VAC		5 VDC	
Frequency	60 Hz			
Transmitter Operating Range		40 - 70 m		
Transmission/receiver time		< 12 s	< 12 s	< 12 s
Wireless Frequency			433.5 MHz	433.5 MHz
Measurement range per phase			50 mA – 120 A	50 mA – 120 A
Measurement accuracy	>90 %			Up to 98%
Storage Capacity				256 kb
Portable display	On mobile apps and web			Yes

**Fig. 5:**
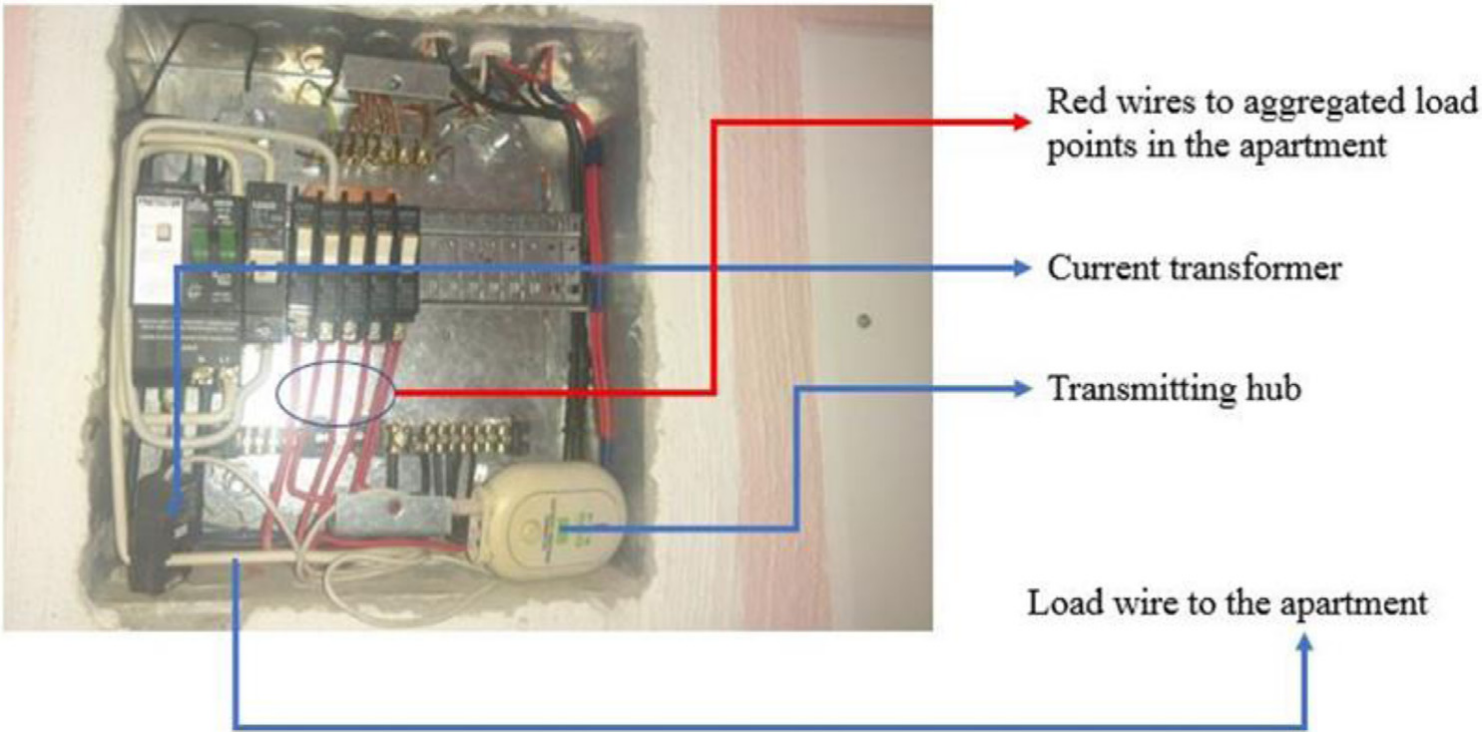
Installation of the current transformer and transmitter.

**Fig. 6:**
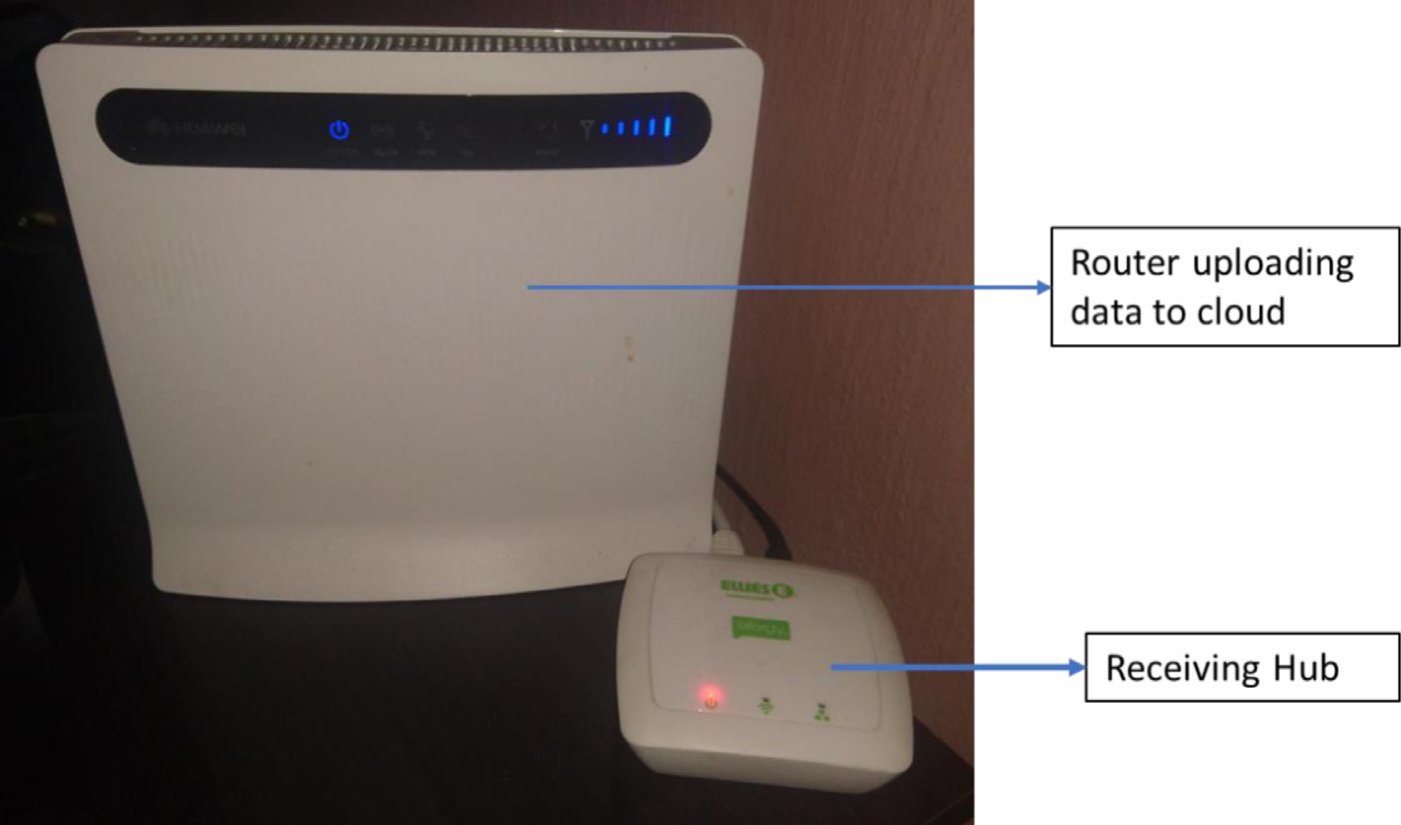
Energy monitor receiver connected to a router.

**Fig. 7:**
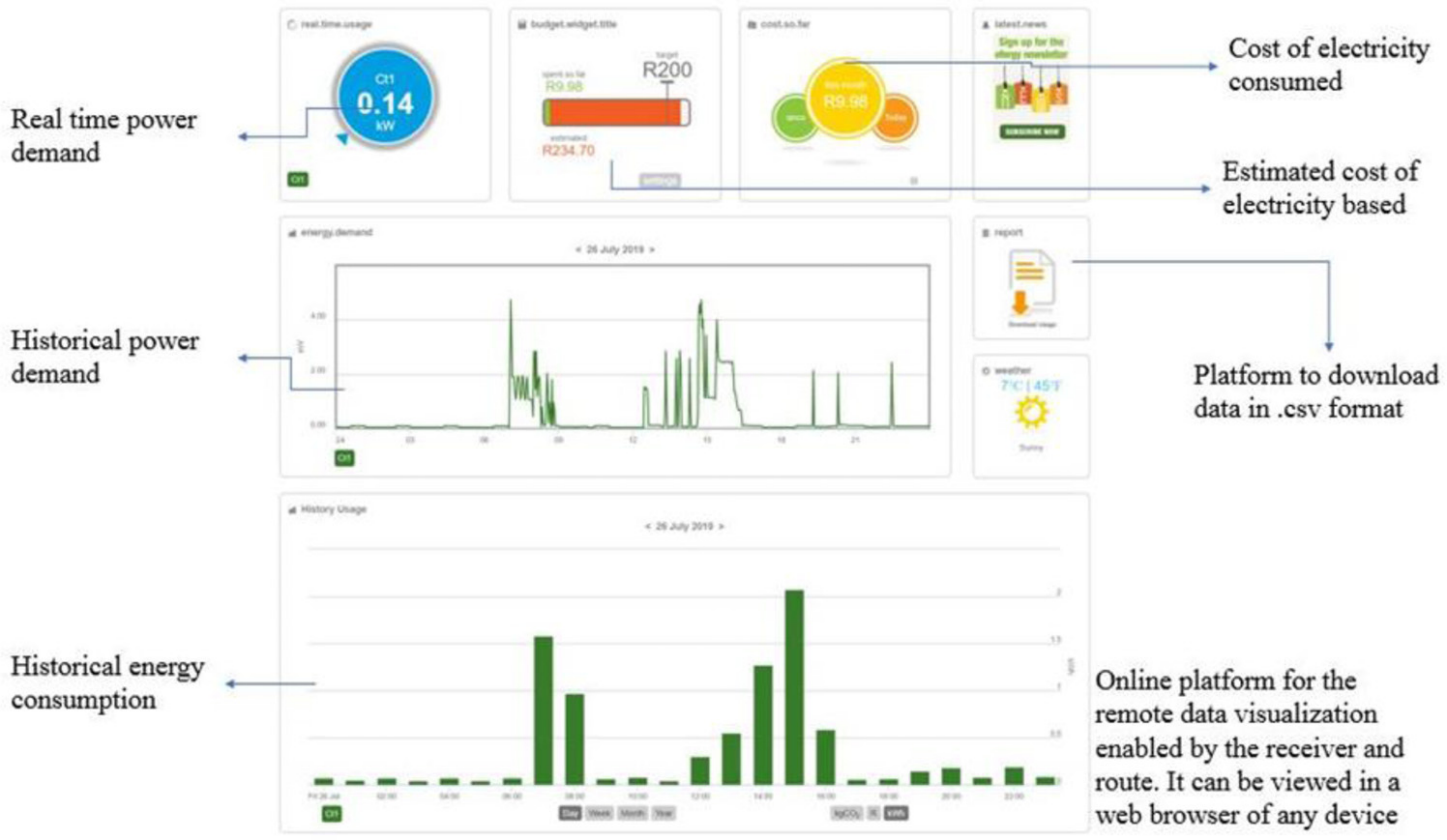
Energy monitor web dashboard showing real-time and historical data.

The current transformer was clamped on the single-phase conductor supplying electricity to the family residence. All three residence has similar distribution board and uses a single-phase 60 Amps supply. A plug pin from the CT scanner was connected to the transmitting hub. The installation at one of the residences is shown in Fig. 5. After the installation of the CT scanner and the transmitter, the internet-based monitoring system was set up and synchronized to the transmitting hub installed on the distribution board. The internet router connected to the transmitter receiving hub as shown in Fig. 6 facilitates data transfer from the transmitter receiving hub to the cloud server of the energy monitor. From the cloud server, the data can be remotely accessed through a web browser or via an app on a smartphone or tablet. A sample of the web-based platform dashboard is shown in Fig. 7. The web-based platform and the smartphone application show the real-time power demand of the entire residence, provide historical power demand profile as well as historical electrical energy consumption.

**Fig. 8:**
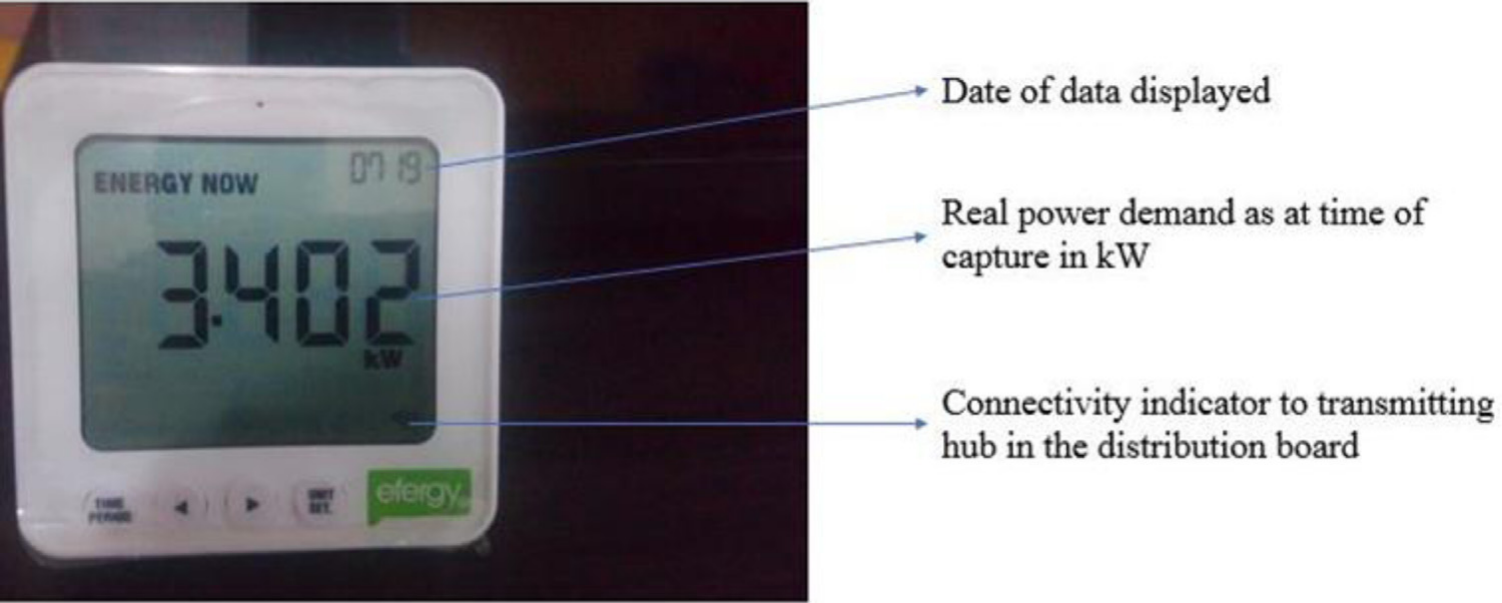
Energy monitor desktop display.

**Fig. 9:**
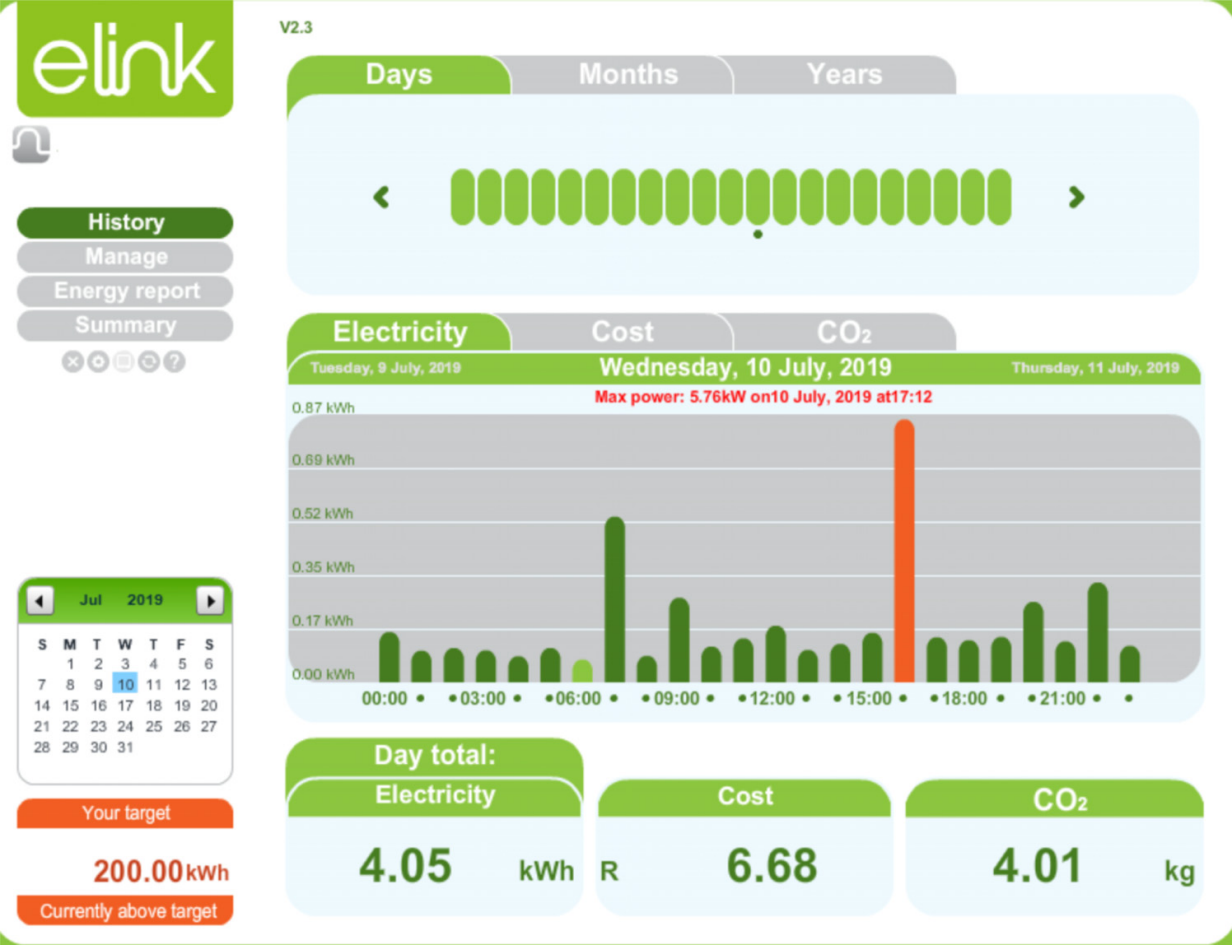
Desktop monitor data visualization and download software.

Aside from the real-time internet-based energy monitor installed, the same CT scanner and transmitter can also communicate to the desktop display monitor in real-time. The desktop monitor is shown in Fig. 8. Data stored in the desktop monitor can be visualised on an e2 classic software as shown in Fig. 9 and downloaded as a .cv file. This allows the occupants who do not have access to the internet to see in real-time the impact of their activities on their electricity consumption. At the end of installation and synchronisation, an accuracy test was conducted. The test involves switching off known loads in Table 2 and the reduction in the captured real-time data was concurrent to the power demand of the respective appliances.

The environmental data were obtained from the closest weather station, Johannesburg Botanical Garden, operated by the South African Weather Service. The weather station is referenced with a climate number of 04758790 at a coordinate of -26.1560, 27.9990 (latitude, longitude) and a height of 1624 m above sea level. The botanical garden is approximately 2 km away from residence A, 11 km from residence B and 60 km from residence C. Details of the environmental data capturing system were not provided.

## Ethics Statement

Ethics approval was not needed for this research. The participant volunteered and agreed to the installation of the energy monitor. The electricity consumption data was shared with the researchers and the household. The data collection did not involve animal experiments.

## CRediT Author Statement

**Roseline Masebinu:** Conceptualization, Methodology, Investigation, Data curation, Writing – original draft, Visualization; **Njabulo Kambule:** Conceptualization, Methodology, Writing – review & editing, Supervision, Project administration.

## Declaration of Competing Interest

The authors declare that they have no known financial interest that could have appeared to influence the data reported in this work.

## Data Availability

Electricity consumption data of a middle-income household in Gauteng, South Africa pre and post COVID-19 lockdown (2019-2021) (Original data) (Mendeley Data).

## References

[1] Masebinu S.O., Holm-Nielsen J.B., Mbohwa C., Padmanaban S., Nwulu N. (2020). Electricity consumption data of a student residence in Southern Africa. Data Brief.

[2] Mustapa S.I., Rasiah R., Jaaffar A.H., Abu Bakar A., Kaman Z.K. (2021). Implications of COVID-19 pandemic for energy-use and energy saving household electrical appliances consumption behaviour in Malaysia. Energy Strategy Rev..

[3] Eskom. (2020, 29 April). *Eskom Loadshedding Schedule*. Available: https://loadshedding.eskom.co.za/

[4] City Power. (2020, 29 April). *Load shedding status*. Available: https://www.citypower.co.za/customers/Pages/Load_Shedding_Downloads.aspx

[5] Efergy, ``E2 wireless electricity monitor,'' Efergy, Ed., ed, 2014.

